# *Salmonella* Typhimurium Infection Leads to Colonization of the Mouse Brain and Is Not Completely Cured With Antibiotics

**DOI:** 10.3389/fmicb.2018.01632

**Published:** 2018-07-18

**Authors:** Debalina Chaudhuri, Atish Roy Chowdhury, Biswendu Biswas, Dipshikha Chakravortty

**Affiliations:** ^1^Microbiology and Cell Biology, Indian Institute of Science, Bangalore, India; ^2^Centre for Biosystems Science and Engineering, Indian Institute of Science, Bangalore, India

**Keywords:** *S*. Typhimurium, brain, antibiotic, neurological abnormalities, infection

## Abstract

*Salmonella* systemic infections claim thousands of lives worldwide even today. Certain cases lead to an infection in the brain culminating in meningitis and associated neurological abnormalities. Multiple reports have indicated neurological manifestations in patients suffering from typhoid fever during the course of infection and afterwards. While the meanderings of *Salmonella* systemic infections are fairly well studied, the flow of events in the brain is very poorly understood. We investigated the colonization of various brain parts by *Salmonella* in mice. It was observed that the bacterium is frequently able to invade various brain parts in mice. Selected mutants namely deletion mutants of key proteins encoded by the *Salmonella* pathogenicity islands (SPIs) 1 and 2 and *ompA* gene were also used to decipher the roles of specific genes in establishing an infection in the brain. Our results suggest roles for the *Salmonella* pathogenicity island (SPI) 1 and outer membrane protein A gene in enabling blood-brain barrier penetration by the pathogen. We further investigated behavioral abnormalities in infected mice and used an antibiotic treatment regime in an attempt to reverse the same. Results show some mice still display behavioral abnormalities and a high bacterial burden in brain despite clearance from spleen and liver. Overall, our study provides novel insights into *S*. Typhimurium's capacity to invade the mouse brain and the effectiveness of antibiotic treatment on behavioral manifestations due to infection. These observations could have important implications in understanding reported neurological manifestations in typhoid patients.

## Introduction

*Salmonella enterica* serovar Typhimurium is a Gram-negative, facultative anaerobe which causes a systemic infection in mice that resembles typhoid fever caused by *S. enterica* serovar Typhi in humans. Infection by *Salmonella* Typhimurium results in a self-limiting gastroenteritis in humans. Till today, infections caused by various species and serovars of the genus *Salmonella* continue to be threats, especially in developing countries (Majowicz et al., 2010; Marathe et al., 2012).

Typhoid fever is commonly characterized by headache, stomach pain and a sustained high fever. In rare cases, typhoid fever patients have been found to exhibit neurological abnormalities (Dewan et al., 2009; Joshi et al., 2011; Szabo et al., 2013; Talukdar et al., 2013). A very high mortality rate, more so in infants, is associated with *Salmonella* infections of the brain (Drevets et al., 2004). Treatment success rates remain alarmingly poor in such cases with relapses adding to the complexity of the situation. Those that survive have been reported to suffer from visual and auditory impairments, cerebral palsy and mental retardation (Kavaliotis et al., 1994; Drevets et al., 2004). While the fundamentals of the systemic infection scenario are well elucidated, the effect of typhoid fever on the brain is a fairly unexplored domain till date.

Central nervous system infections are either fatal or leave lasting neurological damage. While extensive efforts have gone into the understanding of how extracellular pathogens gain entry into the central nervous system or the brain, very little research has focussed on the scenario with intracellular pathogens (Huang and Jong, 2001; Koedel et al., 2002). The mode of translocation of pathogens across the blood-brain barrier could be intercellular, trans-cellular, cell-mediated or some other novel mechanism (Huang and Jong, 2001; Jin et al., 2001).

In the current study, we have used mice (host) infected with *Salmonella* Typhimurium (*S*. Typhimurium) in an attempt to mimic typhoid fever in humans. We investigated the correlation of *S*. Typhimurium burden in the host brain with behavioral abnormalities using two strains of mice: Balb/c and C57BL6. *Salmonella* Pathogenicity Islands (SPIs) 1 and 2 play the most prominent roles in infection (Marcus et al., 2000). Hence, we have also probed the roles of these two pathogenicity islands in brain invasion. In addition, we have identified a role for the outer membrane protein A (ompA) gene in S. Typhimurium brain invasion. Further, using a cell-based system, we have assessed invasion capacities of *S*. Typhimurium in neuronal cells, the most relevant cell form in the mammalian brain. Most importantly, we used the physiological antibiotic treatment regime to understand persistence of the infection in brain post-treatment. To the best of our knowledge, this is the first study reporting the effect of antibiotic treatment upon *S*. Typhimurium burden in mouse brain.

## Results

### *S*. Typhimurium is capable of crossing the blood-brain barrier and reaches various brain tissues

**Figure 1:**
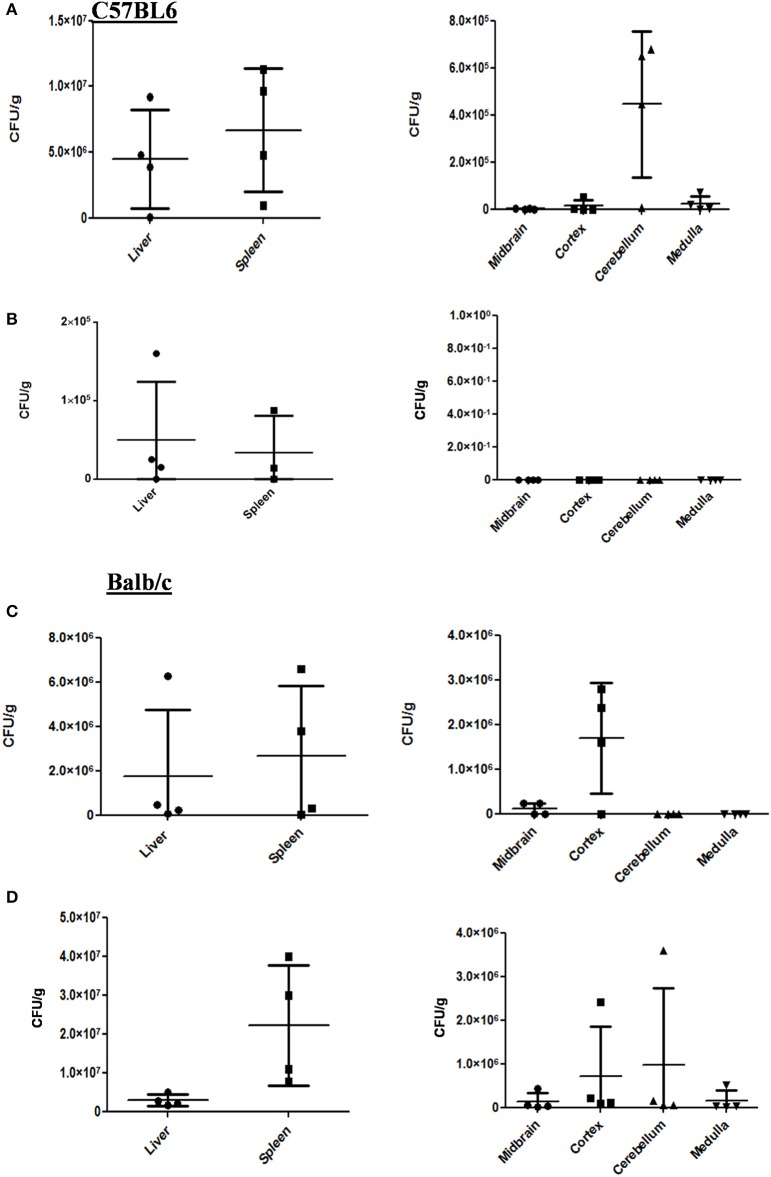
*S*. Typhimurium can invade various parts of murine brain via oral route. **(A)** The CFU/g for spleen, liver and brain parts of C57BL/6 mice infected with 10^7^ CFU of *Salmonella* Typhimurium by oral gavage. The mice were dissected on 5th day post-infection. **(B)** The CFU/g for spleen, liver and brain parts of C57BL/6 mice infected with 10^8^ CFU of *Salmonella* Typhimurium by oral gavage. The mice were dissected on 3rd day post-infection. **(C)** The CFU/g for spleen, liver and brain parts of Balb/c mice infected with 10^7^ CFU of *Salmonella* Typhimurium by oral gavage. The mice were dissected on 5th day post-infection. **(D)** The CFU/g for spleen, liver and brain parts of C57BL/6 mice infected with 10^8^ CFU of *Salmonella* Typhimurium by oral gavage. The mice were dissected on 3rd day post-infection. *n* = 4 mice per group and representative of experiment repeated 5 times (*N* = 5) for Balb/c and (*N* = 3) three times for C57BL6.

Two strains of mouse Balb/c and C57BL6 were subjected to oral gavage with varying doses of *S*. Typhimurium. After designated time-points, the mice were sacrificed and the bacterial concentration in four parts of the brain viz. cortex, cerebellum, midbrain, and medulla were analyzed. The spleen and liver bacterial loads were also determined to authenticate infection (Figures 1A–D). We found that the bacteria could reach one or more parts of the brain at both 10^7^ and 10^8^ dosage albeit with variations amongst mice strains as well as individual mice. In case of C57BL6 mice, 3 days post-infection with 10^8^ bacteria; we could not observe a significant bacterial burden in any brain part.

**Figure 2:**
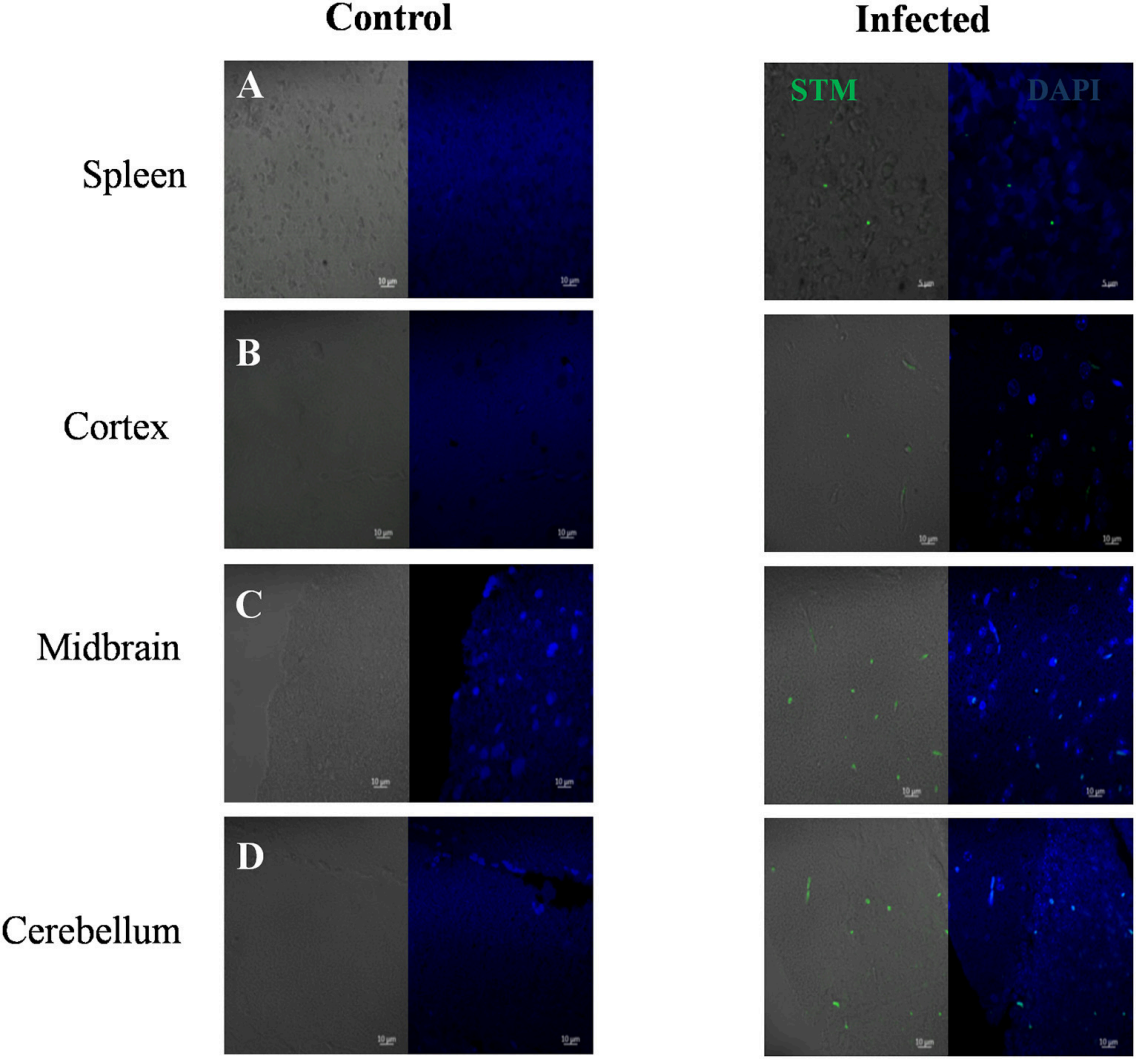
Immunohistochemistry showing *Salmonella* in various brain parts. Mice (Balb/c) spleen and brain parts were dissected and fixed on 5th day. Sections were obtained using a microtome and immunohistochemistry was carried out using DAPI and anti *Salmonella* O antigen antibody. **(A)** Spleen, **(B)** Cortex, **(C)** Midbrain, **(D)** Cerebellum.

**Figure 3:**
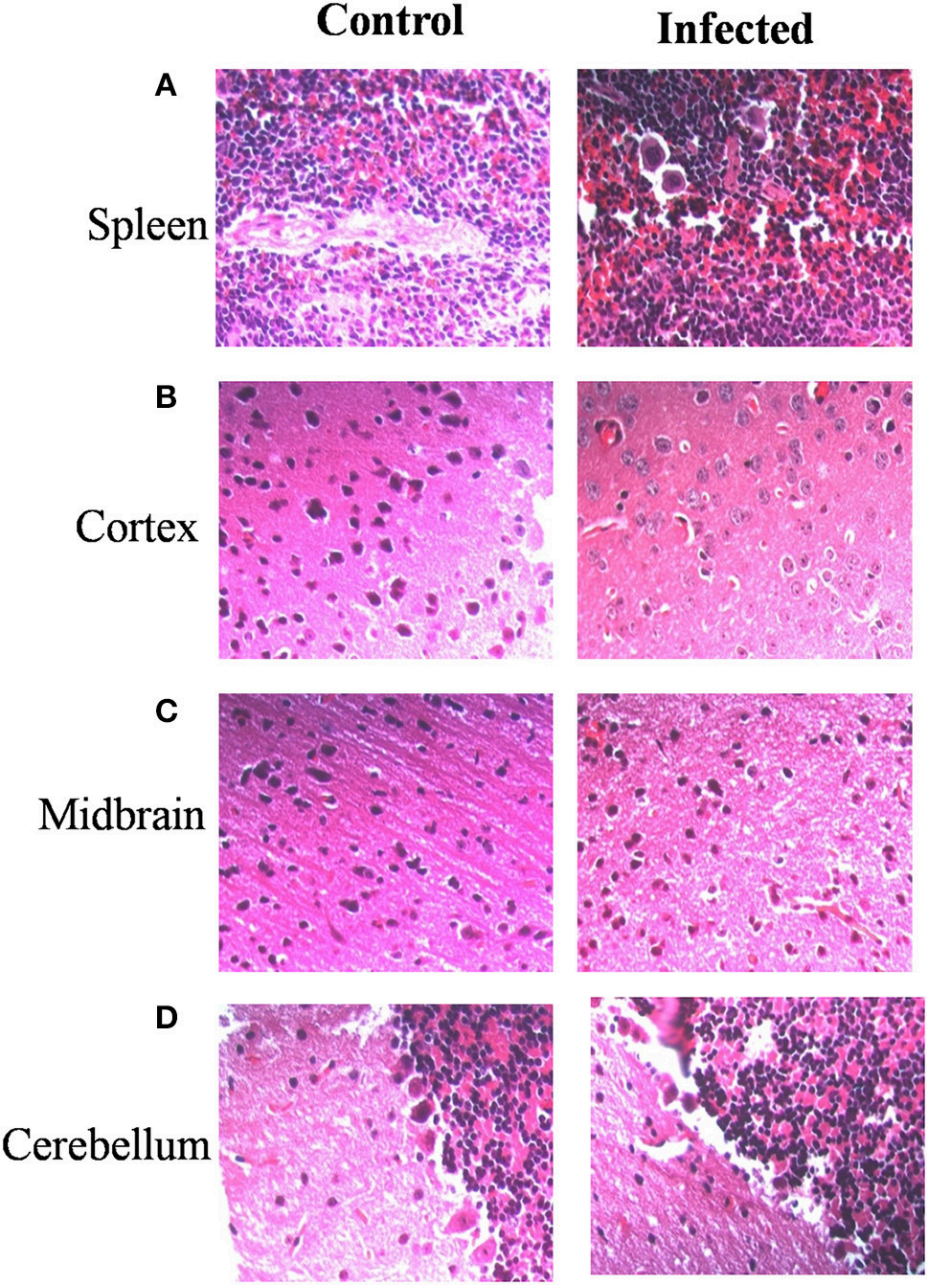
*Salmonella* invasion results in tissue damage in the brain. Mice (Balb/c) spleen and brain parts were dissected and fixed on 5th day. Sections were obtained using a microtome. Haematoxylin and eosin staining was carried out **(A)** Spleen, **(B)** Cortex, **(C)** Midbrain, **(D)** Cerebellum.

Immunohistochemistry using antibodies specific to *Salmonella* O antigen confirmed the presence of bacteria in the cortex, medulla and cerebellum along with spleen (Figures 2A–D). Invasion by *Salmonella* culminates in significant tissue damage in various parts of the brain. As expected, spleens from infected mice were enlarged and showed signs of inflammation (Figure 3A). The midbrain revealed occasional infiltration of neutrophils and congested blood vessels bolstering our previous observations (Figures 3B,C). Furthermore, we could observe a thickened granular layer upon infection in the cerebellum (Figure 3D).

### *S*. Typhimurium can potentially infect neurons

**Figure 4:**
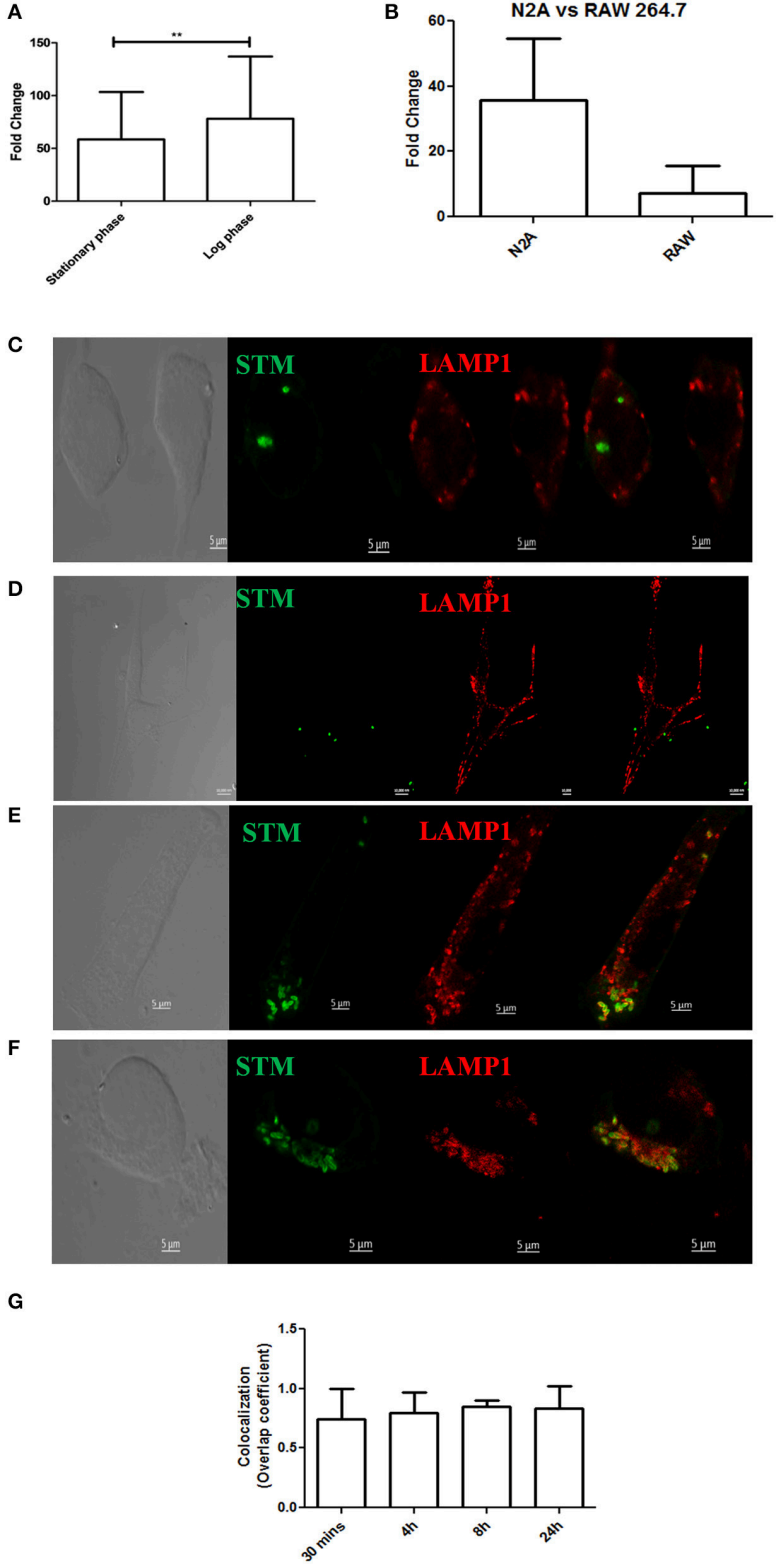
*Salmonella* can invade neuronal cells. **(A)** Fold proliferation of log phase or stationary phase *S*. Typhimurium in N2A cells. **(B)** Comparison between fold proliferation of stationary phase *S*. Typhimurium in N2A cells vs. RAW 264.7 cells. **(C–F)** Confocal images showing N2A cells infected with *S*. Typhimurium at 30 min, 4, 8, and 24 h post-infection respectively. **(G)** Quanitation of colocalization between *S*. Typhimurium and LAMP1. Statistics: Student's *T*-test ^*^P < 0.05. ^***^P < 0.0001.

Considering the presence of bacteria in the cortex, medulla, cerebellum and midbrain, we were curious to know whether *S*. Typhimurium could infect neurons. We used a murine neuronal cell line Neuro2A (N2A) as a model and carried out invasion and intracellular survival assays. Results indicated that both stationary phase and log phase *S*. Typhimurium can very successfully invade and proliferate massively within the neuronal cell line (Figure 4A) and at a rate much higher than in other cells like macrophages (Figure 4B). Immunofluorescence studies indicated the presence of S. Typhimurium in neurons from early through late time points of infection (Figures 4C–F). Cellular staining with the SCV marker LAMP1 revealed that in neurons too, *Salmonella* mostly stays in vacuoles (Figures 4C–G).

### *S*. Typhimurium virulence factors in brain invasion

A majority of pathogenic characteristics of *Salmonellae* can be attributed to one or more of its virulence factors such as the SPIs, virulence plasmids, flagella and others. Some encode secretion systems while others code for effectors and a variety of virulence factors. Two most prominent pathogenicity islands are *Salmonella* pathogenicity island (SPI) 1 and 2. Both these islands code for type three secretion systems. While SPI-1 sends effectors across the host cell membrane and has a prominent role in epithelium invasion, SPI-2 translocates effectors across the SCV membrane and promotes intracellular replication. In trying to understand whether the SPIs can spy into the host brain, we used various mutants that are deficient in either forming the T3SS or in an important effector protein.

**Figure 5:**
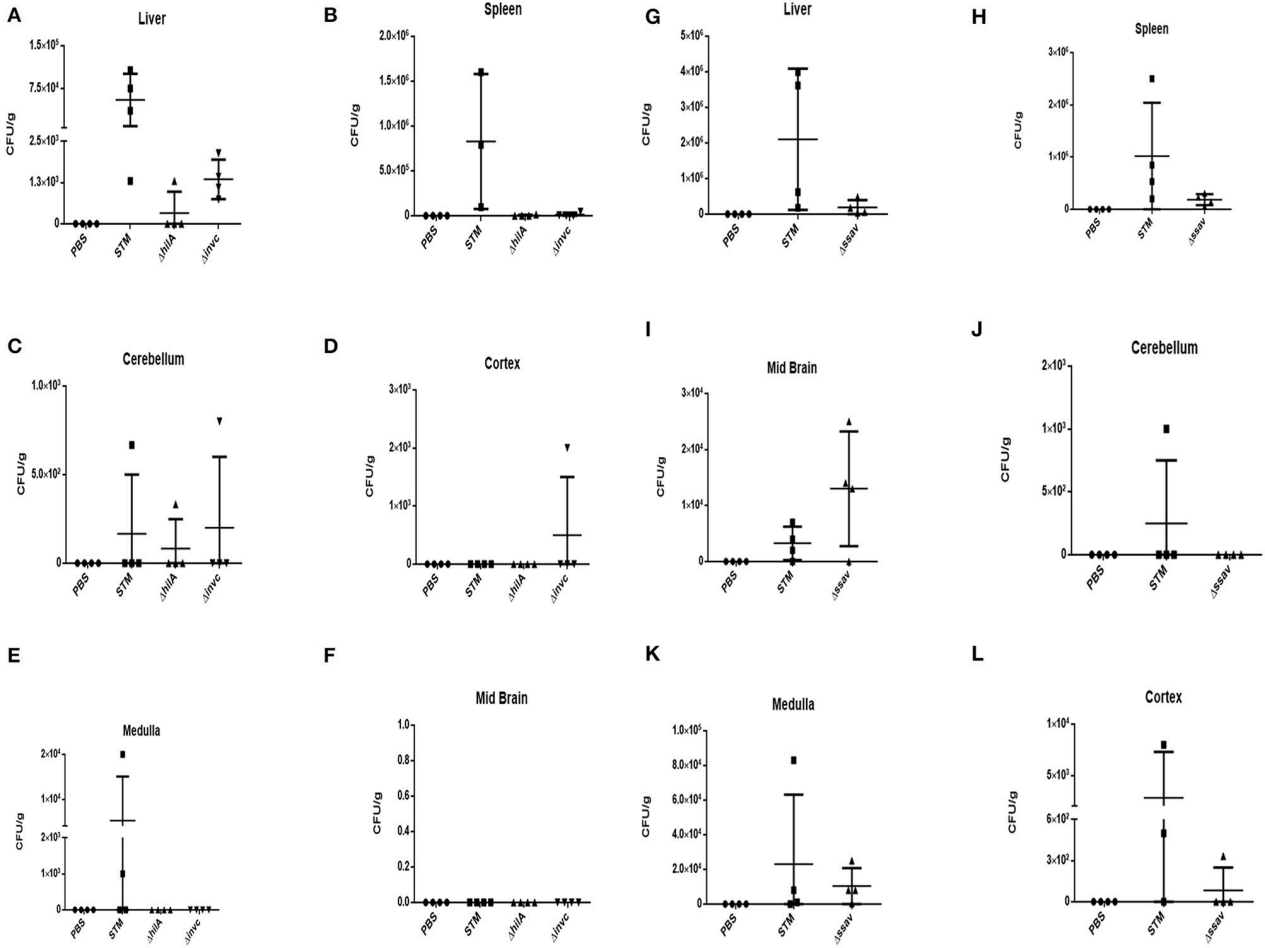
Role of SPI-1 and SPI-2 in brain invasion. CFU burden in **(A)** Liver, **(B)** Spleen, **(C)** Cerebellum, **(D)** Cortex, **(E)** Medulla, **(F)** Midbrain upon oral infection of Balb/c with 10^8^ CFU using SPI-1 mutants. CFU burden in **(G)** Liver, **(H)** Spleen, **(I)** Midbrain, **(J)** Cerebellum, **(K)** Medulla, **(L)** Cortex upon oral infection of Balb/c with 10^8^ CFU using SPI-2 mutants. Representative of *n* = 4 mice per group and *N* = 2.

We found that SPI 1 mutants such as STM Δ*invC* and STM Δ*hilA* were able to cross the blood-brain barrier and reach various parts of the brain, albeit at a lower number which can be attributed to their lesser proliferative capacities in general (Figures 5A–F). The results suggest that an intact SPI-1 aids in effective translocation of the bacteria across the blood-brain barrier. However, their presence in various parts of the brain despite deletions in important SPI-I genes indicates the importance of other factors in blood-brain barrier penetration.

We checked STM Δ*ssaV*, which is a SPI-2 mutant, and found very efficient translocation to reach the brain (Figures 5B,G–H). SPI-2 is primarily expressed inside the SCV and hence it is probable that this island does not play a role in reaching the brain (100% of the mice showed the presence of STM Δ*ssaV* in the brain). These results indicate that an unrelated and unknown dedicated mechanism besides SPI-1 and essential for crossing the blood-brain barrier exists in *S*. Typhimurium. This intriguing question led us to explore various candidates for a role in penetrating the blood-brain barrier.

**Figure 6:**
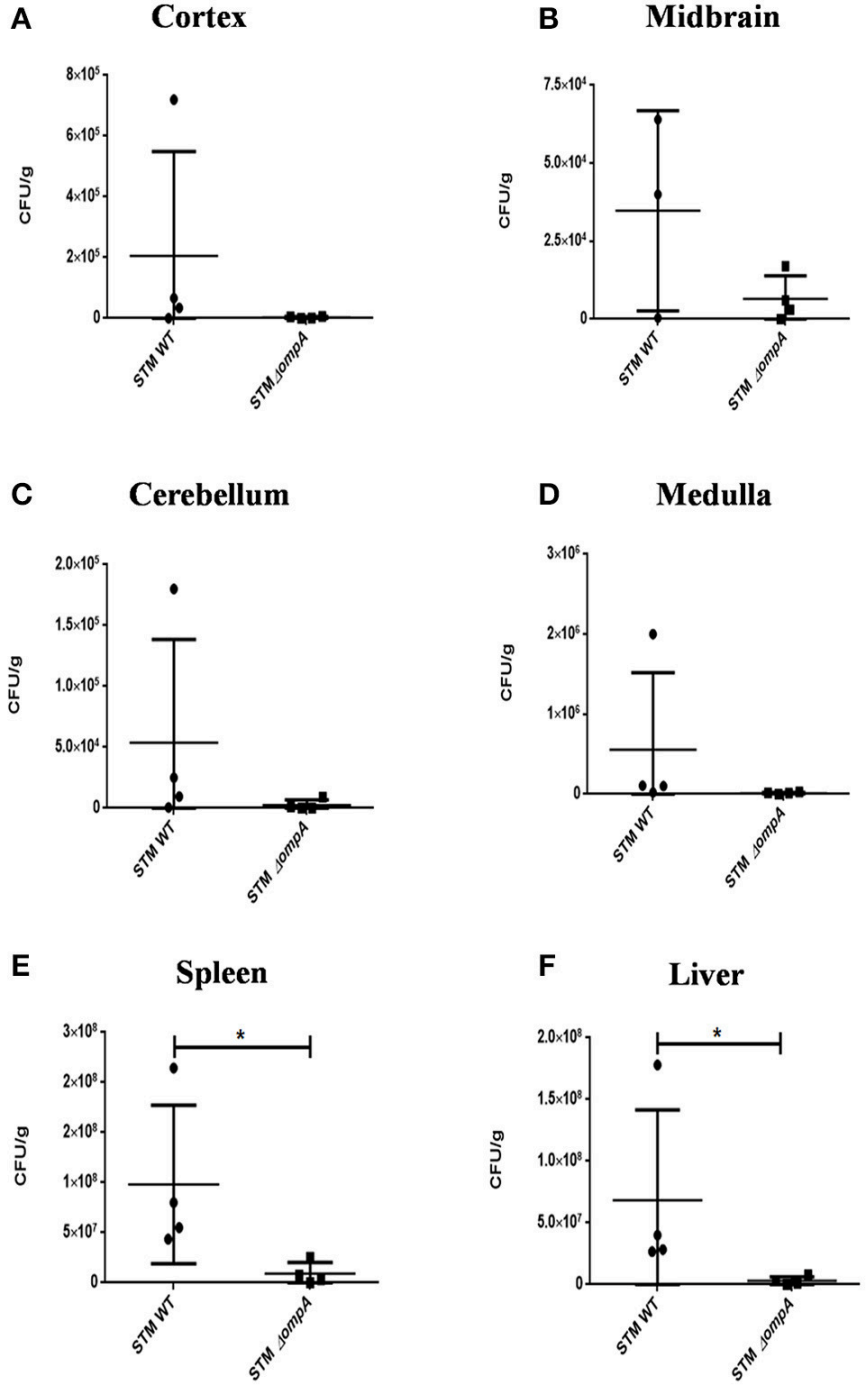
Role of ompA in brain invasion. CFU burden in **(A)** Cortex, **(B)** Midbrain, **(C)** Cerebellum, **(D)** Medulla, **(E)** Spleen, **(F)** Liver upon oral infection of C57BL6 with 10^7^ CFU. [Representative of *n* = 4 mice per group and No. of independent experiments (*N*) = 2].

**Figure 7:**
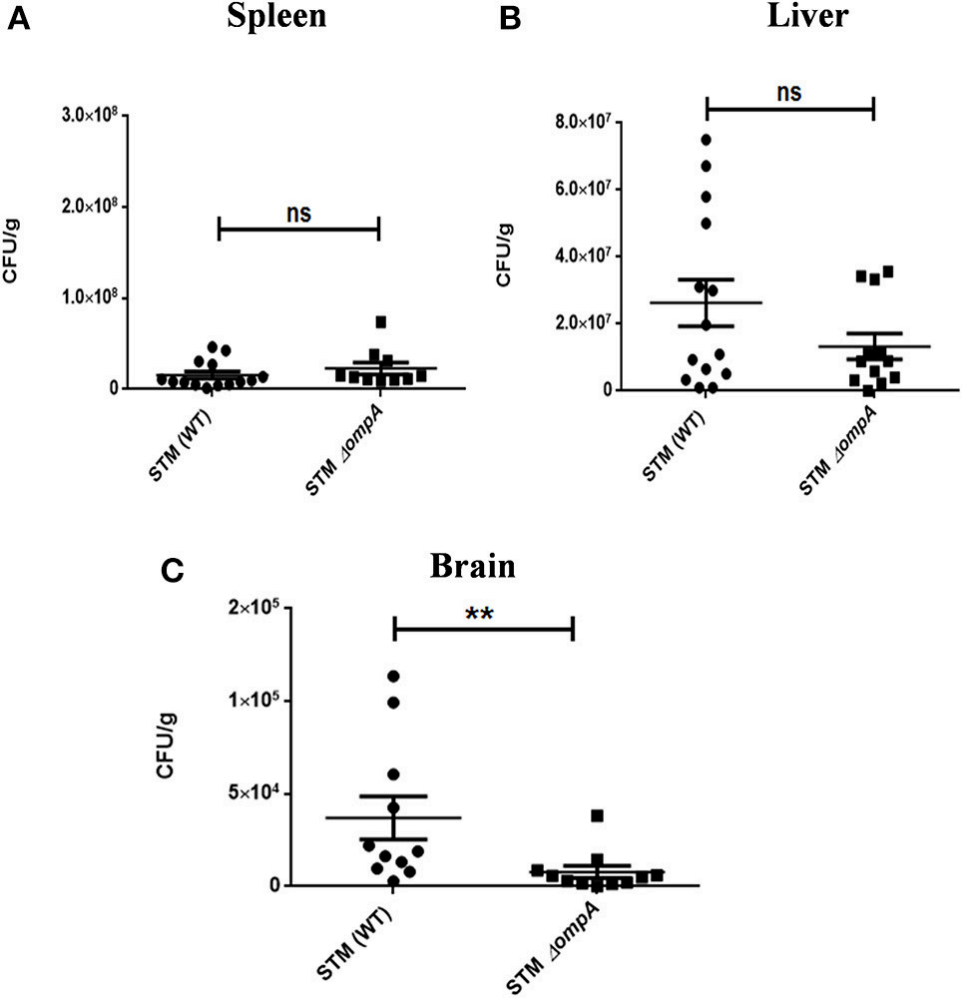
Role of ompA in brain invasion. CFU burden in **(A)** Spleen **(B)** Liver **(C)** Brain upon intraperitoneal infection of C57BL6 with 10^4^CFU. Error bars represent mean ± SEM. Compiled data from three independent experiments (*N* = 3) with 5 mice per group (*n* = 5). Key: ns:not significant, ^**^P < 0.05. Student's *T*-test and Mann Whitney *U*-post-test.

Outer membrane protein A (OmpA) is a constituent protein of the outer membrane of several Gram negative bacteria. Interactions between proteins on the outer membrane of bacteria would conceivably be essential for binding and invasion through the blood-brain barrier. We thus asked whether an outer membrane protein OmpA is essential for blood-brain barrier penetration in case of *S*. Typhimurium. We created a deletion strain with the ompA gene knocked out via one step deletion, and infected mice at a dose of 10^7^ via the oral route. We observed a reduction in bacterial burden in the cortex, cerebellum, midbrain and the medulla (Figures 6A–D). Since the bacterial burden in liver and spleen also showed lower levels (Figures 6E,F), we further used the intraperitoneal mode of infection to bypass the intestinal route. Upon intraperitoneal infection, we observed a significant decrement in the bacterial concentration in mouse brain between wild-type S. Typhimurium and STM Δ*ompA* whereas the bacterial concentrations were similar in liver and spleen (Figures 7A–C).

### Neurological manifestations in infected mice

**Figure 8:**
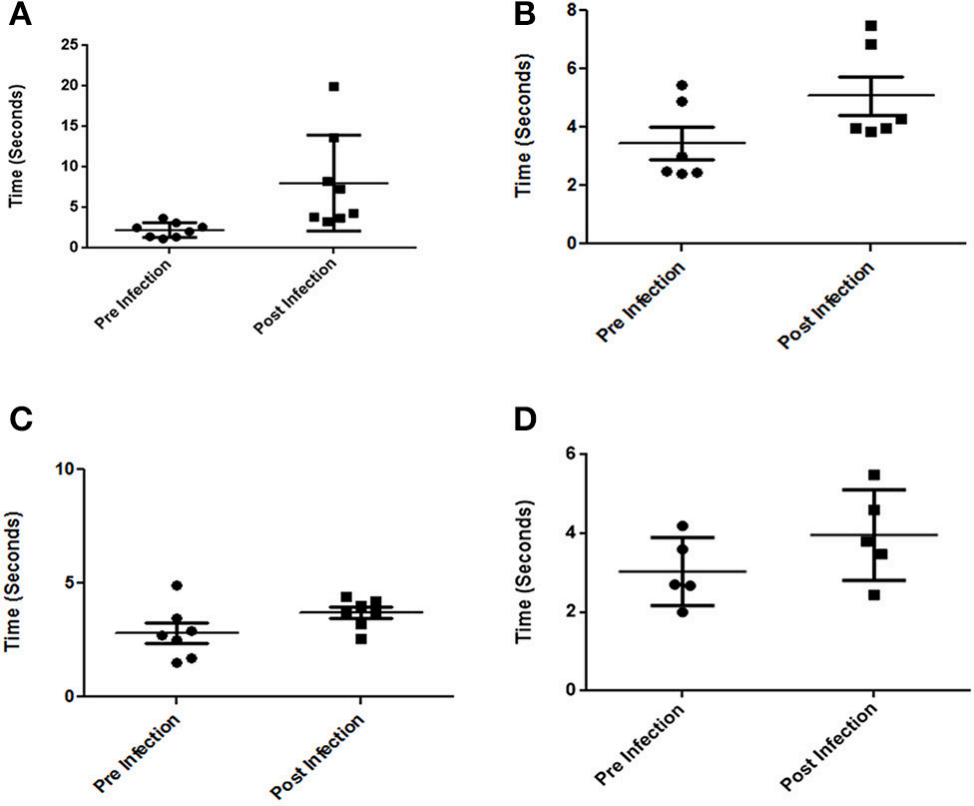
Behavioral changes in mice infected with S. Typhimurium. **(A,B)** Time taken by Balb/c mice to reach a platform in water pre and post-infection with 10^7^ and 10^8^ CFU respetively. **(C,D)** Time taken by C57BL6 mice to reach a platform in water pre and post-infection with 10^7^ and 10^8^ CFU respectively. **(***n* = 4 mice per group and no of independent experiments *N* = 2). Student's *T*-test and Mann Whitney *U*-post-test. ^*^P < 0.01, ^**^P < 0.001.

We have consistently observed that infected mice occasionally develop prominent neurological abnormalities such as rolling and rotatory motions. We carried out water-maze experiments to estimate the time taken by infected mice to reach a platform in water. All mice were first trained for the task following which their performances were recorded both pre and post-infection. We observed a significant increase in the time taken by certain mice infected at either 10^7^ or 10^8^ CFU dosage orally, to remember and swim to the dedicated platform with a few absolutely unable to do so. (Figures 8A–D). We could not however, directly correlate the bacterial load in any particular brain part among those tested, with the behavioral deficiencies.

### Antibiotic treatment cured neurological abnormalities in some but not all mice

**Figure 9:**
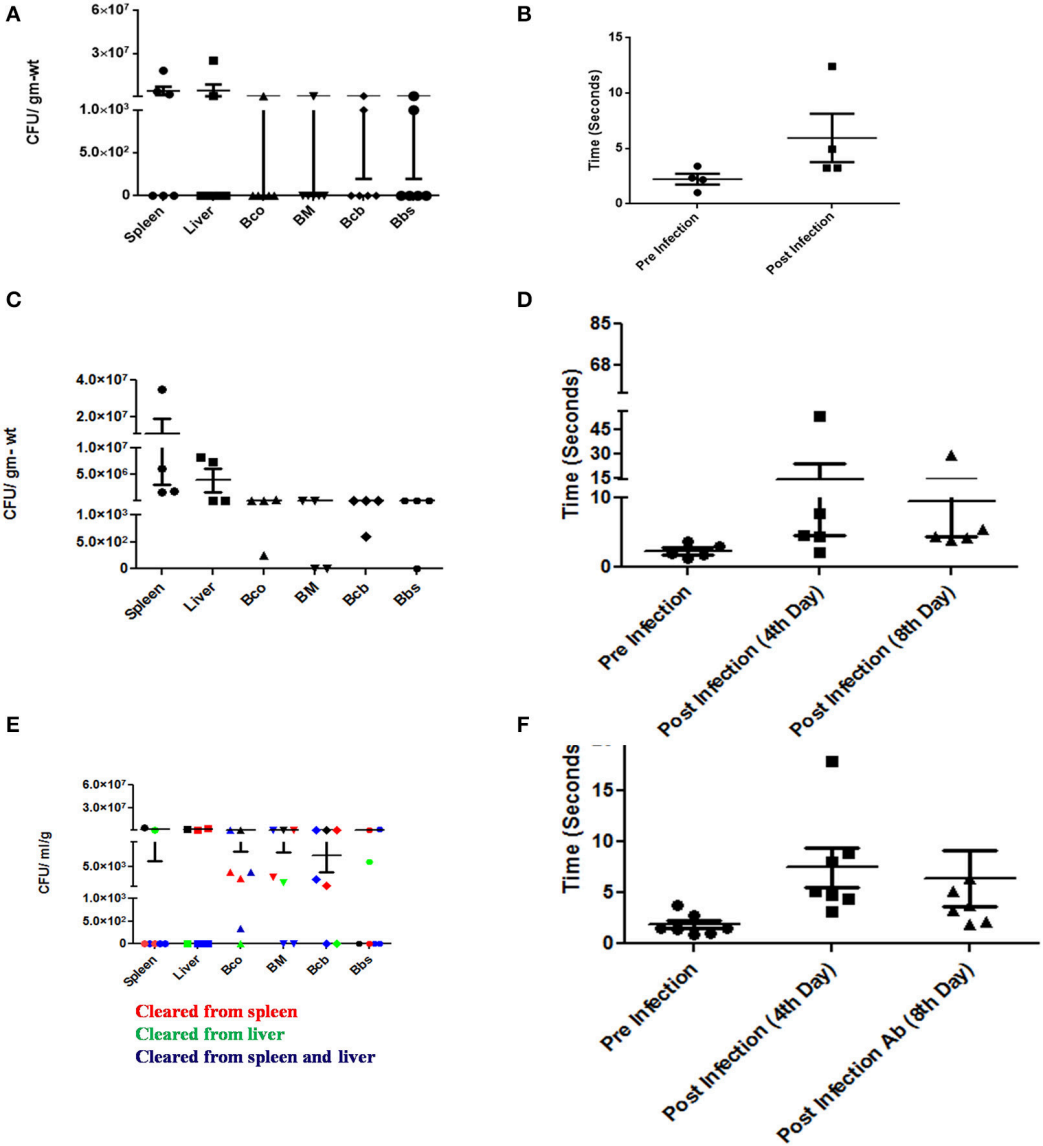
Neurological manifestations of *Salmonella* brain invasion and effect of antibiotics. **(A)** CFU burden in various organs and brain upon oral infection with 10^7^ CFU and dissected on 5th day. **(B)** Time taken by the mice in **(A)** to swim to a platform in a water maze experiment pre and post-infection. **(C)** CFU burden in various organs and brain upon oral infection with 10^7^ CFU and dissected without antibiotic. **(D)** Time taken by the mice in **(C)** to swim to a platform in a water maze experiment pre and different stages post-infection. **(E)** CFU burden in various organs and brain upon oral infection with 10^7^ CFU and dissected post-antibiotic treatment. **(F)** Time taken by the mice in **(E)** to swim to a platform in a water maze experiment pre and different stages post-infection. Each point in the plots represents an animal. Error bars represent mean ± SEM. Compiled data from two independent experiments (*N* = 2) with 4 mice per group (*n* = 4). Few animals died prior to the designated test days. Statistics: Student's *T*-test and Mann-Whitney *U*-post-test. Key: Bco-Cortex, Bcb:Cerebellum, BM-Midbrain, Bbs-Medulla. Ab-Antibiotic.

Typhoid patients manifest neurological symptoms during and even years after the disease incidence. This led us to investigate the behavior of infected mice after antibiotic (Figure S1) treatment and the corresponding bacterial burden. While some mice showed a restoration in their ability to find the water maze platform, there were animals which were still strikingly incapable of performing the task. Moreover, bacteria were still present in the brains of even the better performing mice (Figures 9A–F).

These results could have serious implications in the general neurological health of the host in the long run. It would be an interesting question to see whether at later stages, neurological abnormalities resurface in these mice essentially simulating abnormal neurological conditions encountered in humans much after they have been “cured” of typhoid.

## Discussion

*Salmonella* infections are frequent in developing nations around the world. Neuro-psychiatric symptoms related to typhoid fever have been observed for years (Khosla et al., 1977; Inoue et al., 1995; Ali et al., 1997). Alarmingly, multidrug resistant typhoid incidences have also been found to be associated with neurologic manifestations (Lutterloh et al., 2012).

While we have looked at *S*. Typhimurium infection in mice which mimics human typhoid fever, the effect of non-typhoidal infections in this regard cannot be ignored. Tracking and data collection regarding self-limitting gastrointestinal diseases would be considerably more challenging to understand the interaction with brain in that regard. Consequently, reports are expected to be limiting (Rizek et al., 2013). Recently, Bauler et al. have tested the importance of host NRAMP1 in *S*. Typhimurium burden in the brain (Bauler et al., 2017).

Our results corroborate the observations made by Bollen et al. (2008) using intranasal and intraperitoneal infections and those of Wickham et al. (2007). Further, we observed an enhanced thickness of the granular layer in infected cerebellum. Enhanced granular layer thickness has been associated with age related degeneration and might be an important sign of neurological deficit (Zhang et al., 2006) The present study provides a detailed understanding of *S*. Typhimurium genes like *ompA* and SPI-1 genes, that take part in mediating host brain invasion. Here, we show that ompA is essential for brain invasion by *S*. Typhimurium. Apart from its structural roles, OmpA also serves a pathogen-associated molecular pattern in bacteria belonging to Enterobacteriaceae (Jeannin et al., 2002). Besides its other roles, OmpA has been shown to be an important player in blood-barrier penetration of a number of bacteria especially *E. coli* (Khan et al., 2003; Maruvada and Kim, 2012). OmpA of *C. sakazakii* has been reported to promote invasion of brain endothelial cells in association with fibronectin (Nair et al., 2009). *Enterobacter sakazakii* requires OmpA for invasion of brain endothelial cells via induction of microtubule condensation (Singamsetty et al., 2008) Furthermore, the effect of antibiotic treatments on the brain bacterial burden has tremendous physiological relevance in today's world with emerging antibiotic resistance adding to the problem.

The implications of *Salmonella* burden in various parts of the brain could be far reaching. Proliferation of the pathogen in various brain parts could be associated with an enormous variety of neurological symptoms. Moreover, we found a very high proliferation capacity in neuronal cell lines bolstering our hypothesis that the neurological manifestations post-infection are effects of possible cell death or modulation in the brain. Further, cellular endotoxins are known to induce inflammation in substantia nigra dopaminergic neurons. TNF-α, IFN-γ, and IL-6 are secreted by astrocytes and microglia upon LPS stimulation (Herrera et al., 2000; Liu et al., 2000). Hippocampal slice cultures were found to undergo neurodegeneration in presence of *Salmonella* LPS (Johansson et al., 2005). Interestingly, sepsis has been found to make the blood-brain barrier leaky (Brandtzaeg et al., 1989) possibly leading to a greater infiltration of endotoxins. Effects mediated by endotoxins could therefore lead to a battery of symptoms in context of the neurological manifestations. Glial cells could also serve as a niche for *Salmonella* in the brain. Conceivably, *Salmonella* would be less proliferative in glial cells, however, they may act as a niche harboring persister populations.

With the average life-span of humans getting extended every decade and the complexities in modern lifestyles, we are also witnessing an increase in the incidences of neurological abnormalities ranging from depression, anxiety, bipolar disorders to diseases like Parkinson's disease and Alzheimer's disease.

In view of the enhanced incidences, the possible causes such as external pathogens are of outstanding importance. With genetic predisposition to such conditions on one hand, such external agents as bacterial infections could be viewed with heightened seriousness and in turn avoided. A recent report has drawn connections between Alzheimer's disease and *Salmonella* (Kumar et al., 2016). Their study revealed that the functional aspect of the β amyloid protein is to offer protection from microbial attacks. Plaques were observed to be formed around the bacterial cells thereby entrapping them. Very conceivably, this phenomenon could easily steer toward an unpleasant outcome for the host in the absence of a very tight regulation. This arena calls for a much greater understanding to actually unveil the cause and effect scenario of intracellular bacterial pathogens in the brain and allow active alleviation of the avoidable causes of neurological manifestations.

## Materials and methods

### Bacterial strains, media, and growth conditions

Wild type (WT) *S. enterica* serovar Typhimurium Strain 14028 and deletion strains were grown in Luria broth with appropriate antibiotics (if any) under shaking conditions at 37°C. Overnight cultures prepared from inoculation of a single colony from a fresh plate were used for infecting mice at the mentioned dosage. Either overnight culture or 1:33 subcultured bacteria were used for cell-line experiments.

### Generation of knockout strains

STM Δ*ompA* were generated by one-step chromosomal gene activation method as described before (Datsenko and Wanner, 2000; Zhang et al., 2006). Briefly, the kanamycin resistance gene cassette was PCR amplified from pKD4 plasmid using specific primers which also carried sequences homologous to the flanking region of the target gene. The resultant insert was electroporated into WT STM pKD46. The knockouts were confirmed using both primers against the gene of interest as well as Kan^R^ cassette internal primers. STM Δ*invC*, STM Δ*hilA*, and STM Δ*ssaV* were kind gifts from Prof. Michael Hensel.

### Eukaryotic cell maintenance

The murine macrophage cell line RAW 264.7 & mouse neuroblastoma cell line Neuro2a were maintained in DMEM (Sigma-Aldrich) supplemented with 10% FCS (Gibco) at 37°C with 5% CO_2_.

### Intracellular survival assay

A monolayer of respective cells was infected with either overnight or 0.3 O.D._600_ cultures (mentioned accordingly) at an MOI of 10. Bacterial attachment to host cells was enhanced by centrifuging at 700–1,000 rpm for 5 min. After 25 min of infection, cells were treated with gentamicin at 100 μg/ml for 1 h. Following this, cells were maintained in DMEM containing 25 μg ml-1 gentamicin through the entire experiment. Infected cells were lysed using 0.1% Triton X-100 at 2 and 16 h post-infection and appropriate dilutions spread on Salmonella-Shigella (SS) agar plates.

Fold Proliferation:
CFU at 16 h was divided by CFU at 2 h to obtain fold replication of intracellular bacteria.
Percent invasion:
Percent invasion was calculated as: (no. of intracellular bacteria/no. of input bacteria) × 100.

### Immunofluorescence

For immunofluorescence analysis, RAW 264.7 or Neuro2A cells, seeded at a density of 0.5–1 × 10 5 cells per coverslip, were infected with bacteria at MOI 10. At indicated time points post-infection, cells were washed with PBS and fixed with 3.5% paraformaldehyde for 10–15 min. The cells were then washed with PBS and incubated with specific primary antibody against mouse lysosome-associated membrane protein 1 (LAMP1) (rat anti-mouse LAMP1; Developmental Studies Hybridoma Bank), and/or *S*. Typhimurium (anti- *Salmonella* O antigen) as per the experiment diluted in blocking buffer containing 2% BSA and 0.01% saponin. Post-washes, cells were then incubated with appropriate secondary antibody conjugated with fluorophores. Coverslips were mounted on a glass slide containing mounting medium. Images were acquired using confocal laser scanning microscope (Zeiss LSM 710 or Zeiss LSM 880). Images were analyzed using the Zen software provided by Zeiss.

### Animal experiments

The animal experiments were carried out in accordance with the approved guidelines of the animal ethics committee at Indian Institute of Science, Bangalore, India (Registration No: 48/1999/CPCSEA). Two strains of mice, 4–6 weeks old were used viz. BALB/c and C57BL/6.

The mice were orally gavaged with 10^7^ cells/ ml and dissected on 5th day post-infection or 10^8^ cells/ ml (with the respective bacterial cultures depending on the experiment) and dissected on 3rd day post-infection.

For intraperitoneal infections, mice were injected with 10^4^ bacterial cells into the peritoneal cavity and dissected 3rd day post-infection.

Behavioral studies were carried out with the mice both pre and post-infection by water maze test. Infection was by oral gavage with 10^7^ cells/ ml and dissection on 5th day post-infection.

In case of the antibiotic experiments, ciprofloxacin was administered orally at a dose of 100 mg/ Kg body weight from 5th to 8th day post-infection. The dose was determined based on literature (Brunner and Zeiler, 1988) and the length of treatment determined on the survival of the control group receiving placebo. The 5th day dissection was carried out to ensure proper infection of the cohort. The main comparison of bacterial burden was done between the placebo treated group and the antibiotic treated group at the end of the entire experiment. Behavioral tests were carried out pre and post-infection as well as post-antibiotic treatment.

In all the experiments, the mice were sacrificed and organs isolated under asceptic conditions. The samples were weighed, homogenized using a bead-beater and plated on Salmonella-Shigella agar to obtain the CFU burden in the organs. The CFU/g was then calculated based on the organ weights obtained.

In all experiments, each group contained 4–6 mice and 2–6 independent experiments were performed unless otherwise mentioned.

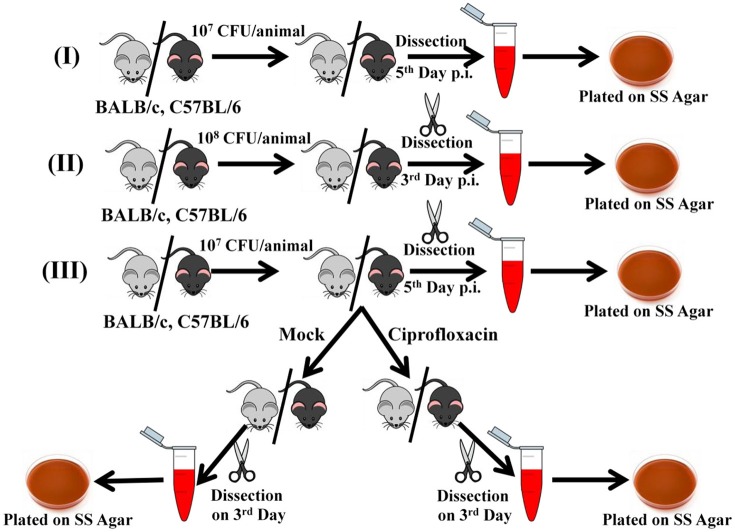



### Histological studies

Mouse organs were isolated aseptically and fixed with paraformaldehyde. Following dehydration, sections embedded in paraffin were obtained using a microtome. Sections were deparaffinised in xylene and rehydrated through a series of alcohol (90, 70, 50, 30%; 1 min each) and finally immersed in distilled water for 5 min. Haematoxylin-eosin staining was performed for some sections while others were processed for staining with antibody against *Salmonella* O antigen. An Alexa fluor 488 conjugated secondary antibody was used to visualize the bacteria under Zeiss LSM 710 along with DAPI staining.

### Statistical analysis

The data were analyzed using either Students' *t*-test and/or Mann-Whitney *U*-test with Graph Pad prism 6 software.

## Author contributions

DC designed and carried out all experiments. BB helped in the cell line experiments. AR carried out animal experiments with DC. DC and DiC wrote the manuscript.

### Conflict of interest statement

The authors declare that the research was conducted in the absence of any commercial or financial relationships that could be construed as a potential conflict of interest.
