# Supplementary figures and images for: Salmonella Typhimurium Infection Leads to Colonization of the Mouse Brain and Is Not Completely Cured With Antibiotics
Source: Front Microbiol. 2018 Jul 18;9:1632. doi: 10.3389/fmicb.2018.01632 (PMC6058050; doi:10.3389/fmicb.2018.01632)

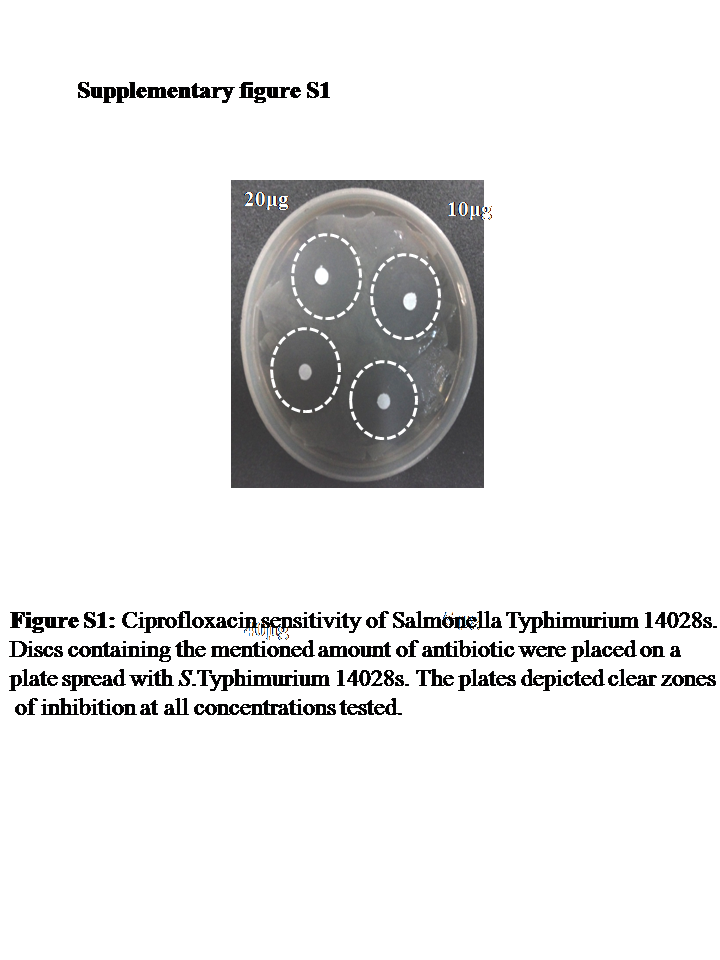

Supplement: Supplementary file 1 [file Image_1.TIF]
